# Evaluation of the patency rate and endothelialization of a poly-ε-caprolactone, nanofiber sheet-based vascular graft using a rat abdominal aortic implantation model

**DOI:** 10.3389/fsurg.2024.1464155

**Published:** 2024-11-21

**Authors:** Yuta Kikuchi, Naohiro Wakabayashi, Daikelly I. Braghirolli, Patricia Pranke, Hiroyuki Kamiya, Kyohei Oyama

**Affiliations:** ^1^ Department of Cardiac Surgery, Asahikawa Medical University, Asahikawa, Japan; ^2^ Sapporo Cardiovascular Clinic, Department of Cardiovascular Surgery, Sapporo, Japan; ^3^ Hematology and Stem Cell Laboratory, Faculty of Pharmacy, Universidade Federal Do Rio Grande Do Sul, Porto Alegre, Brazil

**Keywords:** vascular graft, poly-ε-caprolactone, electrospinning, nanofiber, endothelialization

## Abstract

**Introduction:**

The global increase in cardiovascular diseases has resulted in an augmented development of artificial small-caliber vascular grafts used in bypass graft surgeries, such as coronary and distal artery bypass graft surgeries. However, no consensus exists regarding the best method for creating vascular grafts. Poly-ε-caprolactone (PCL) is a biocompatible and biodegradable material that has been widely studied as a scaffold for tissue regeneration, inclusive of vascular grafts. In this study, a vascular graft was created from a PCL nanofiber sheet (PCL graft), and the performance thereof was examined using a rat abdominal aortic implantation model.

**Methods:**

The PCL nanofiber sheets were created using an electrospinning machine. These nanofiber sheets were rolled up. Glue was applied between layers using a PCL solution to create a PCL nanofiber vascular graft, with an inner diameter of 1 mm. PCL grafts with 7 mm length were implanted into the abdominal aorta of rats. Thereafter, the patency was determined by pulsating blood flow from the hemiresection site of the distal aorta of the graft anastomosis, and endothelialization was examined using hematoxylin and eosin and immunofluorescent staining methods.

**Results:**

The patency rate of the PCL graft at 2 weeks was 57.1% (12 of 21 cases), which is not satisfactory as a small-caliber vascular graft. Patent cases, however, revealed a CD31-positive endothelial cell layer in the inner lumen and autologous cell infiltration into the scaffold, indicating autologous vessel-like regeneration. By contrast, the occluded cases showed disassembly of the nanofiber layers; and the inner layers folded into the middle of the lumen. This observation suggested that the disassembled inner layer of the PCL graft disturbed the blood flow and triggered occlusion.

**Conclusions:**

PCL grafts can exhibit autologous vessel-like regeneration; nonetheless, regarding patency, grafts made from rolled-up PCL nanofiber sheets have structural weaknesses. Further improvements are required to achieve a long-term and high patency rate for PCL grafts.

## 1 Introduction

Globally, an increase in cardiovascular diseases is driving the demand for small arterial bypass surgeries, such as coronary and distal artery bypass graft surgeries (1, 2). Autologous vessels are the only option for bypass grafts, because currently available artificial vascular grafts with small diameters, conventionally <4 mm, are easily occluded due to thrombosis. However, the number and quality of autologous vessels are limited in clinical settings, due to the preexisting conditions of patients who undergo revascularization surgery. Therefore, the development of small artificial vascular grafts with high patency rates is a requisite (3).

Poly-ε-caprolactone (PCL) is a synthetic, biodegradable polymer produced from cyclic ester caprolactone monomers. Characterized by biodegradability and a high biocompatibility, renders it useful in the field of tissue engineering (4, 5). Electrospun PCL nanofibers have been extensively studied as structural scaffolds for tissue regeneration, particularly in the field of vascular tissue engineering (6). However, no consensus exists regarding the best method for creating vascular grafts. In previous studies (7, 8), PCL nanofibers have been electrospun onto a mandrel, using high-voltage direct-current power. Thereafter, the mandrel has been pulled away. However, in our preliminary study, the pull-away process of the mandrel destroyed the PCL graft (unpublished data); therefore, an alternative method is required. Consequently, in the present study, a vascular graft was created by rolling an electrospun PCL nanofiber sheet. Moreover, the performance of this nanofiber sheet-based PCL graft was tested using a rat abdominal aorta implantation model.

## 2 Materials and methods

### 2.1 PCL graft preparation using the rolling-up method

**Figure 1:**
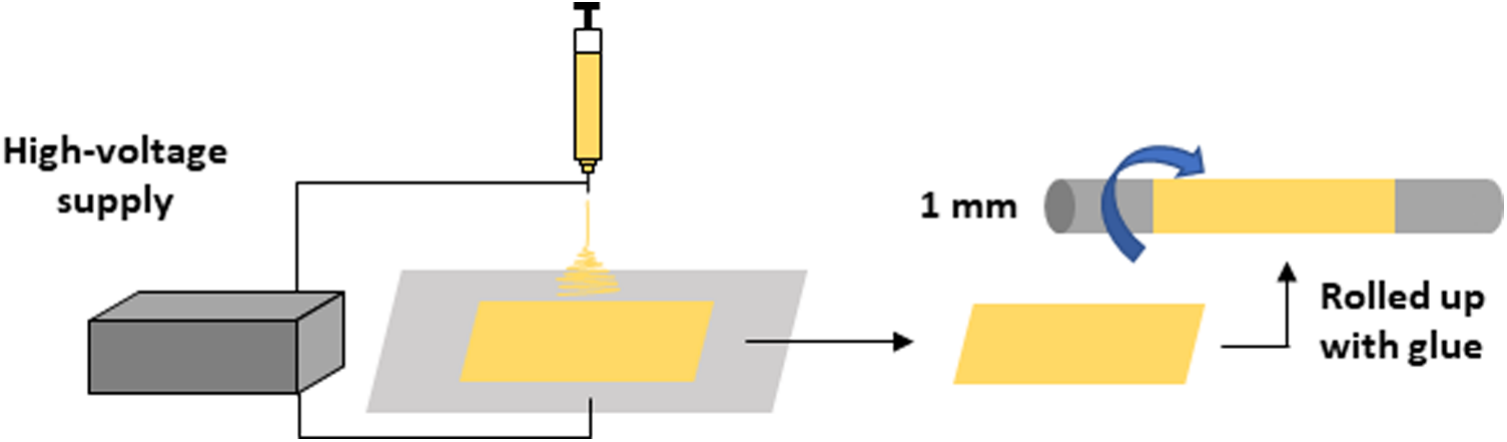
Preparation of the poly-ε-caprolactone graft is depicted.

A PCL nanofiber sheet was produced by electrospinning, as has been described in a previous study (9). PCL (10% w/v) was dissolved in tetrahydrofuran (THF) and N,N-dimethylformamide (DMF) (7:3). The solution was stirred overnight to achieve complete homogenization. The polymeric solution was loaded into a 1 mL syringe with a 0.7 mm internal diameter needle. The needle was placed at a 20 cm distance from the collector plate, and electrospinning was conducted as a flow rate of 0.019 mL/min and a voltage of 19 kV at room temperature (20°C). The resulting PCL nanofiber sheet was sterilized with ultraviolet light for 2 h and subsequently rolled up on an axle of stainless steel that was 1 mm-in-diameter. The layer was glued using a PCL solution (20% w/v in THF:DMF 7:3). The morphologies of the fibers were analyzed using scanning electron microscopy (SEM; JEOL JSM-6060, Tokyo, Japan). Images were obtained at an accelerated tension of 10 kV after the samples were coated with a thin layer of gold using a sputter coater (Bal-Tec SCD 050, Tokyo, Japan). The average diameter of the fibers was determined by measuring 30 fibers per each image (*n* = 3), using software ImageJ 1.383 (NIH, Bethesda, MA, USA). The resulting PCL graft was formed by random fibers (average diameter 427 ± 195 μm) and 1 mm inner diameter (9) (Figure 1).

### 2.2 Graft implantation and harvesting

All protocols concerning the use of animals were approved by the Institutional Animal Care and Use Committee of Asahikawa Medical University (reference number R5-027-02).

Male Wistar rats (Charles River, Kanagawa, Japan) were anesthetized in a small chamber filled with 5% isoflurane for induction, and anesthesia was maintained with a mask ventilation of 2%–3% isoflurane. Vital monitors were not employed during and after this procedure. The abdominal areas were shaved and scrubbed with alcohol gauze. A midline abdominal incision was performed. The abdominal aorta was isolated by dissecting the periaortic tissues and the tissues around the inferior vena cava using cotton swabs to create enough space for clamping the aorta. After clamping the caudal part of the left renal artery and bifurcation of the aorta, the abdominal aorta was transected. The PCL graft with 7 mm length was anastomosed via end-to-end running sutures using 10-0 polypropylene. The aorta was subsequently unclamped. The aortic clamping time averaged 15 min. After hemostasis, the abdominal incision was closed. The rat was placed in a normal cage, and its return to full consciousness was confirmed. Subsequently, the rats were provided with standard food and water. No antiplatelet or anticoagulant drugs were administered during follow-up as following previous studies (10–12).

Fourteen days postoperatively, heparin was intraperitoneally administered to the rats. After 15 min, they were euthanized with an isoflurane overdose. Graft patency was determined by pulsating blood flow from the hemiresection site of the distal aorta of the graft anastomosis. The grafts were washed with phosphate buffered saline (PBS) and fixed with 4% paraformaldehyde (PFA) via perfusion from the cardiac apex. Thereafter, the grafts were harvested.

### 2.3 Histological evaluation

Histological analysis was performed, as has been previously described (13, 14). Harvested grafts were fixed in 4% PFA overnight and washed with PBS the following day. The grafts were cut into three transverse sections, embedded in an optimal cutting temperature (OCT) compound (Sakura Finetek Japan, Tokyo, Japan), and stored at −80°C. The frozen samples were subsequently sliced (5 µm-thick) using a cryostat (Leica CM3050S; Leica BIOSYSTEMS, Tokyo, Japan) at −20°C and placed on a glass slide for histological analysis.

Hematoxylin and eosin (H&E) staining was performed to detect morphological changes in the graft lumen. The frozen sample was washed with PBS for 5 min to remove the OCT compound and subsequently immersed in a hematoxylin solution (FUJIFILM, Tokyo, Japan) for 4 min, followed by a 6-min wash with running water. Thereafter, the sample was immersed in 1% eosin solution (FUJIFILM, Tokyo, Japan) for 2 min, and washed thrice with 70% ethanol. The resulting samples were encapsulated in Marinol 750 (Muto Puro Chemical Co., Tokyo, Japan) and examined.

For immunofluorescence (IF) staining, heat-induced epitope retrieval at 98°C for 1 h was performed, using a tris-ethylenediaminetetraacetic acid (EDTA) buffer (containing 10 mM Tris base, 1 mM EDTA, and 0.05% Tween 20; pH 9.0). The sample was blocked using 1% bovine serum albumin in PBS before antibody reactions. Primary antibody, anti-CD31 (1:200, #AF3628; R&D Systems, Minneapolis, MN, USA); and secondary antibody, Alexa Fluor 488 conjugated anti-goat immunoglobulin G (IgG), were used in this study. Hoechst 33342 (FUJIFILM, 346-07951, Tokyo, Japan) was used for nuclear staining. The HE and IF images were captured using an all-in-one fluorescence microscope (BZ-X810; Keyence, Osaka, Japan).

## 3 Results

### 3.1 Autologous tissue engraftment of PCL graft in patent case

**Figure 2:**
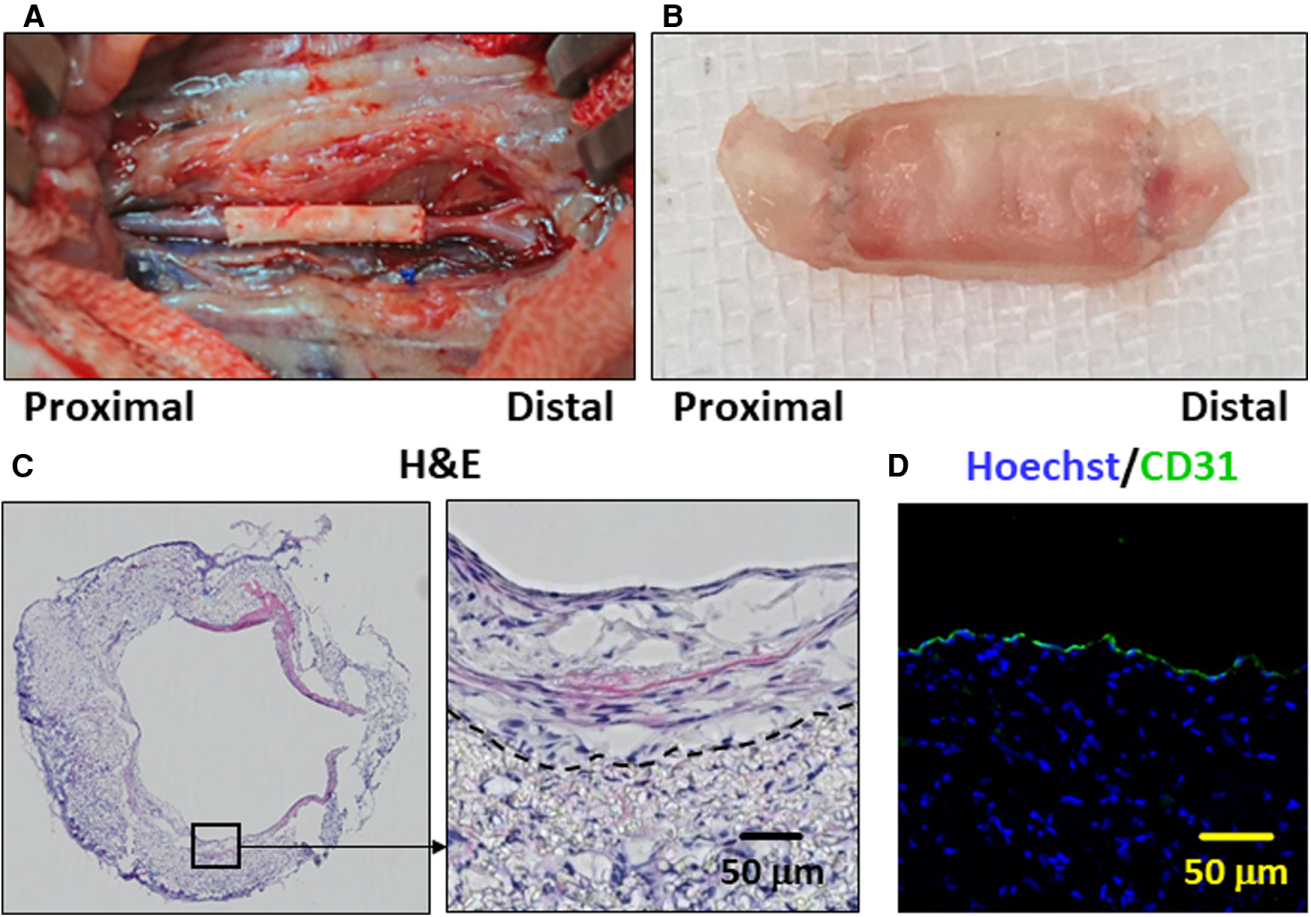
The poly-ε-caprolactone (PCL) graft implantation and histological images of the patent cases are presented. **(A)** Representative image of the PCL graft after implantation in the rat abdominal aorta. **(B)** Representative image of the PCL graft explanted after 2 weeks. **(C)** Representative hematoxylin and eosin (H&E)-stained histological image is depicted of the PCL graft which has remained patent for 2 weeks. The dotted line indicates the border between the PCL scaffold and engrafted autologous cells on the luminal side. **(D)** Representative immunofluorescence staining image is shown. CD31- and Hoechst-stained histological images are in green and blue, respectively.

A PCL graft was produced by the rolling-up method (Figure 1 in the Materials and Equipment), and the performance thereof was tested as a vascular graft *in vivo* (Figures 2A,B). The patency rate at 2 weeks was 57.1% (12 of the 21 cases). An examination was performed to determine whether the PCL grafts exhibited autologous cell engraftment (Figures 2B,C). IF staining revealed a CD31-positive cell layer in the inner lumen (Figure 2D). Furthermore, Hoechst staining was observed inside the PCL scaffold, suggesting autologous tissue-like regeneration with endothelial cells.

### 3.2 Disassembled nanofiber layers of PCL graft in occluded cases

**Figure 3:**
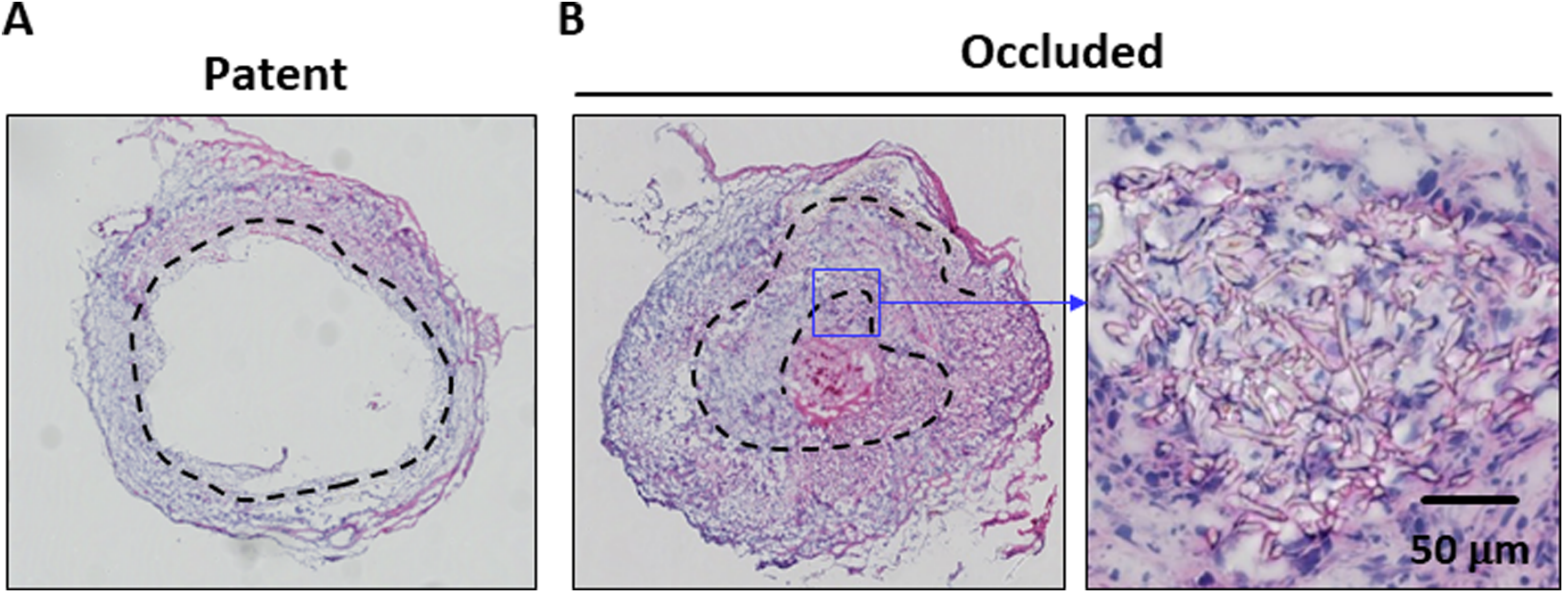
Comparative images of the patent and occluded cases are shown. Hematoxylin and eosin (H&E)-stained histological images of the patent **(A)** and occluded **(B)** poly-ε-caprolactone (PCL) grafts are shown. The dotted line indicates the location of the PCL scaffold. The occluded PCL graft shows disassembly of the layers and is folded into the middle of the lumen.

Subsequently, the potential causes of PCL graft occlusion were investigated. H&E-stained histological images showed that the PCL grafts of the patent cases had tightly assembled scaffold layers (Figure 3A). However, most of the occluded cases showed disassembly of the nanofiber layers; and the inner layers folded into the middle of the lumen (Figure 3B). This observation suggested that the disassembled inner layer of the PCL graft disturbed the blood flow and triggered occlusion. Thus, PCL grafts made from rolling PCL sheets are postulated of being susceptible to disassembly, resulting in occlusion.

## 4 Discussion

In this study, PCL vascular grafts were produced by rolling up electrospun PCL nanofiber sheets. Performance thereof was tested *in vivo*, using a rat abdominal aortic implantation model. Consistent with the results of previous reports, the PCL graft in this study exhibited autologous tissue-like regeneration, characterized by endothelial cell formation in the lumen and autologous cell engraftment into the PCL nanofiber scaffold. Thus, the ability of the PCL nanofiber scaffold to regenerate vessel-like tissues was highly reproducible, demonstrating that PCL is a promising material for vascular grafts.

However, the patency rate of the PCL grafts in this study was approximately 57%; and approximately half of the cases were occluded within 2 weeks. Compared with the patent cases, where the PCL nanofiber layers were tightly assembled to the endpoint, the occluded PCL grafts showed disassembled layers, which was probably due to the method employed to create the PCL graft. Because the PCL grafts were created from PCL nanofiber sheets, one end of the sheet was present on the luminal side of the PCL graft. When gluing the end of the sheet is incomplete, the inside layer flaps are postulated to disturb the blood flow. It has been well described in the clinical literature that blood flow disturbances result in vascular occlusion, due to the activation of coagulation (15). Therefore, an improved gluing method is essential to achieve a better patency rate when creating a vascular graft from a nanofiber sheet. Furthermore, a seamless PCL graft lumen is expected to improve patency. Indeed, several reports exist regarding the creation of PCL nanofiber grafts with seamless lumens, demonstrating longer-term patency than that in the present study (7, 16). This negative result supports the importance of the seamless lumen method for creating PCL nanofibers, based on vascular grafts.

## Data Availability

The raw data supporting the conclusions of this article will be made available by the authors, without undue reservation.
